# Dissociating executive prefrontal substrates following capsulotomy in obsessive-compulsive disorder

**DOI:** 10.1093/brain/awag132

**Published:** 2026-04-16

**Authors:** Yi-Jie Zhao, Hailun Cui, Yingying Zhang, Ruiqin Chen, Luis Manssuer, Haibin Li, Jiechun Zhang, Wenjuan Liu, Bomin Sun, Valerie Voon

**Affiliations:** Institute of Science and Technology for Brain-Inspired Intelligence, Fudan University, Shanghai 201210, China; Clinical Research Center for Mental Disorders, Shanghai Pudong New Area Mental Health Center, School of Medicine, Tongji University, Shanghai 200124, China; Department of Neurosurgery, Huashan Hospital, Shanghai Medical College, Fudan University, Shanghai 200040, China; Department of Psychiatry, University of Cambridge, Cambridge CB2 0QQ, UK; Clinical Research Center for Mental Disorders, Shanghai Pudong New Area Mental Health Center, School of Medicine, Tongji University, Shanghai 200124, China; Institute of Science and Technology for Brain-Inspired Intelligence, Fudan University, Shanghai 201210, China; Clinical Research Center for Mental Disorders, Shanghai Pudong New Area Mental Health Center, School of Medicine, Tongji University, Shanghai 200124, China; Department of Psychiatry, University of Cambridge, Cambridge CB2 0QQ, UK; Department of Psychological Medicine, Zhongshan Hospital, Fudan University, Shanghai 200032, China; Institute of Science and Technology for Brain-Inspired Intelligence, Fudan University, Shanghai 201210, China; Clinical Research Center for Mental Disorders, Shanghai Pudong New Area Mental Health Center, School of Medicine, Tongji University, Shanghai 200124, China; Department of Psychological Medicine, Zhongshan Hospital, Fudan University, Shanghai 200032, China; Department of Psychiatry, Shanghai United Family Pudong Hospital, Shanghai 201206, China; Department of Neurosurgery, Center for Functional Neurosurgery, Clinical Neuroscience Center, Ruijin Hospital, Shanghai Jiao Tong University School of Medicine, Shanghai 200025, China; Clinical Research Center for Mental Disorders, Shanghai Pudong New Area Mental Health Center, School of Medicine, Tongji University, Shanghai 200124, China; Department of Psychiatry, University of Cambridge, Cambridge CB2 0QQ, UK; Key Laboratory of Computational Neuroscience and Brain-Inspired Intelligence (Fudan University), Ministry of Education, Shanghai 200433, China; Zhangjiang Fudan International Innovation Center, Shanghai 201203, China

**Keywords:** Obsessive-Compulsive Disorder, capsulotomy, executive function, functional magnetic resonance imaging

## Abstract

Obsessive-compulsive disorder (OCD) is characterized by widespread executive function impairments linked to disrupted frontostriatal circuits. Ablative therapies such as capsulotomy show promising efficacy in treatment-refractory OCD, yet their effects on executive neural substrates remain poorly understood. This study explores how OCD and capsulotomy influence executive prefrontal function.

Twenty-three post-capsulotomy OCD patients, 30 OCD controls and 32 health controls (HC) were recruited in the study. Post-capsulotomy patients were recruited at least 6 months following surgery to allow for postoperative stabilization. Participants completed three executive function tasks assessing distinct cognitive domains: the Extra-Dimensional Intra-Dimensional (EDID) task testing set-shifting, the N-back task testing working memory and the Flanker task measuring conflict processing. The EDID and N-back tasks were administered during concurrent task-based functional MRI, while the Flanker task provided only behavioural measures.

In the EDID task, OCD capsulotomy patients demonstrated greater post-error flexibility towards ID versus ED shifting relative to both OCD controls and HC, despite exhibiting more ED errors. OCD controls showed less shifts after errors compared with HC, which was not revealed in the capsulotomy group. OCD patients showed less neural differentiation between ED versus ID in lateral and mesial prefrontal regions relative to HC. Capsulotomy was associated with decreased pre-supplementary motor area activity to ED shifts compared with HC, indicating impairments in neural reactivity to ED shifting. No evidence supported the influence of capsulotomy in the N-back or the Flanker tasks. Specifically, both OCD groups showed impaired working memory performance at high memory load along with dysfunction in the whole frontoparietal network. In the Flanker task, impairments in dissociating congruent and incongruent conditions were found in both OCD groups but not in the HC group.

These findings suggest capsulotomy specifically remediates error-monitoring and behavioural flexibility through mesial prefrontal remodelling, while leaving working memory and conflict processing deficits intact. This dissociation implies that cognitive flexibility may represent a state-dependent process amenable to intervention, whereas working memory and conflict processing impairments may reflect trait markers of OCD vulnerability. These results have clinical implications on the relative safety profile of capsulotomy and patient selection for precision neuromodulation.

## Teaser

Zhao *et al.* report that capsulotomy surgery improves error monitoring and behavioural flexibility in individuals with severe obsessive-compulsive disorder, while leaving working memory and conflict processing deficits intact. They suggest that these persistent deficits may be trait markers of the disorder.

## Introduction

Obsessive-compulsive disorder (OCD) is a prevalent and chronic psychiatric condition characterized by intrusive, recurring thoughts and repetitive behaviours related to aberrant frontostriatal circuitry. Surgical interventions such as deep brain stimulation (DBS) and ablation have shown efficacy targeting specific brain structures within this network for severe OCD.^1-3^ A common target in both DBS and ablative surgeries is the white matter anterior limb of the internal capsule (ALIC), which connects key prefrontal and subcortical regions associated with OCD. Converging evidence from DBS surgeries highlight rostral dorsal cingulate fibre tracts traversing the ALIC associated with optimal clinical responses. Similarly, our studies on capsulotomy highlight modulation of aversive processing in this same region.^4-7^

Previous studies have suggested that executive deficits may serve as endophenotypic markers and risk factors for the development of OCD.^8^ A range of deficits are commonly observed in OCD in executive impairments including error monitoring,^9,10^ inhibitory control,^11,12^ set-shifting,^13^ goal-directed control and planning,^14-16^ along with visual memory deficits.^17,18^ A meta-analysis focusing on trait endophenotypes comparing OCD patients and their unaffected relatives highlighted a range of cognitive impairments in inhibition, planning and reward-based decision tasks as trait risk factors and further suggested set-shifting and visual memory impairments observed commonly in OCD also reflected incipient sub-syndromal OCD.^19^ To identify the neural correlates underlying executive impairments, task-based neuroimaging studies in OCD focusing on executive function implicated in set-shifting, inhibition and conflict and error processing (e.g. Wisconsin Card Sorting Test, Stop Signal Task and Stroop) have found abnormal activity in key brain structures of the frontostriatal network, including the anterior cingulate cortex (ACC),^10,20,21^ orbitofrontal cortex (OFC),^10,20^ lateral prefrontal cortex (lPFC)^22-25^ and subcortical regions,^15^ all of which are within the frontostriatal network likely modulated by various surgical interventions.^26^ Studies on the impact of surgical interventions have focused predominantly on behavioural rather than on imaging substrates.^27^ Understanding the impact of capsulotomy on cognitive processes in OCD is crucial to assessing the potential risks and benefits, in addition to aiding patient selection and optimal targeting across neurosurgical procedures in OCD.

In the present study, we selected cognitive tasks known to be impaired in OCD and reflecting differing prefrontal substrates to assess the effect of capsulotomy using task-based functional MRI (fMRI). We have previously shown in this same cohort that OCD capsulotomy modifies hyperactive aversive expectation mediated by downregulation of activity in the dorsal cingulate.^7^ Furthermore, we show that OCD is characterized by impairments in dorsal cingulate and anterior insula activity in differentiating uncertainty which remains unchanged by capsulotomy suggesting a trait effect.^28^ Here, we tested OCD controls (OCD contr), OCD patients post-capsulotomy (OCD cap) and healthy controls (HC) on the Extra-Dimensional Intra-Dimensional (EDID) set-shifting task (testing attentional set shifting), the N-back task (testing working memory) and the Flanker task (testing conflict and adaptation). We hypothesized that the capsulotomy procedure could modify executive functioning and neural activity within the frontostriatal network during executive tasks, potentially explaining changes or stability in performance among OCD patients compared with controls.

## Materials and methods

### Participants

A total of 85 participants (30 OCD contr, 23 OCD cap and 32 HC) were recruited. Each task was completed by a subset of participants (See Supplementary Tables 1–3 for exact numbers and demographic information of participants in each task). All patients who underwent capsulotomy were scanned no earlier than 6 months post-surgery. This timing is based on long-term follow-up studies indicating that the therapeutic outcomes of the procedure typically stabilize after 6 months,^29,30^ while cognitive changes are most frequently observed between 3 and 6 months afterward.^27^ Comorbidities of schizophrenia, bipolar disorder, substance abuse or dependence (except for dependence on nicotine), personality disorder and severe major depressive episode were excluded. Participants with any severe medical, neurological, psychosis symptoms and head injury were also excluded. In the OCD contr group, one participant exhibited a comorbid status with mild generalized anxiety disorder and two participants had mild depressive symptoms. In the OCD cap group, one participant had mild depressive symptoms. Detailed criteria can be found in the Supplementary material as well as in our previous publications.^7,28^ The study was approved by the ethics committee of Ruijin Hospital and written informed consent forms were obtained.

### Stimuli and procedure

#### Extra-Dimensional Intra-Dimensional set-shifting task

The Extra-Dimensional Intra-Dimensional (EDID) paradigm (Fig. 1A), adapted from the attentional set-shifting task used in previous neuroimaging studies,^31^ consisted of two runs with each containing two sections and 14 blocks in total. In each block, participants saw two different shapes, each paired with a different letter, and had to identify the ‘correct’ feature (shape or letter) by pressing left or right on a button box (Fig. 1B). The same feature needed to be selected twice to ensure that participants were adhering to the instructions and the location of the stimuli could change. Each response was followed by immediate feedback (lasting 0.6 s) to help participants learn the correct feature over several trials. In the event that the participant chose two different stimuli, the trial would be disregarded, and a new trial would commence, with the inter-trial interval randomized between 1 and 4 s. Following three consecutive correct trials (considered ‘learned’), the task shifted either extra-dimensionally or intra-dimensionally between the two dimensions (shape or letter). An ID shift was defined as a shift to a new target feature within the same dimension similar to a reversal (e.g. letter ‘S’ to ‘T’). An ED shift was defined as a shift to a new target feature from a different dimension (e.g. letter ‘I’ to shape ‘triangle’). When a shift occurred, participants received incorrect feedback if they chose the correct feature from the previous block. This prompted them to learn a new sorting rule based on the feedback provided. Each section of the task consisted of three ED shifting blocks and three ID shifting blocks, resulting in a total of four acquisition blocks, 12 ED blocks and 12 ID blocks.

**Figure 1:**
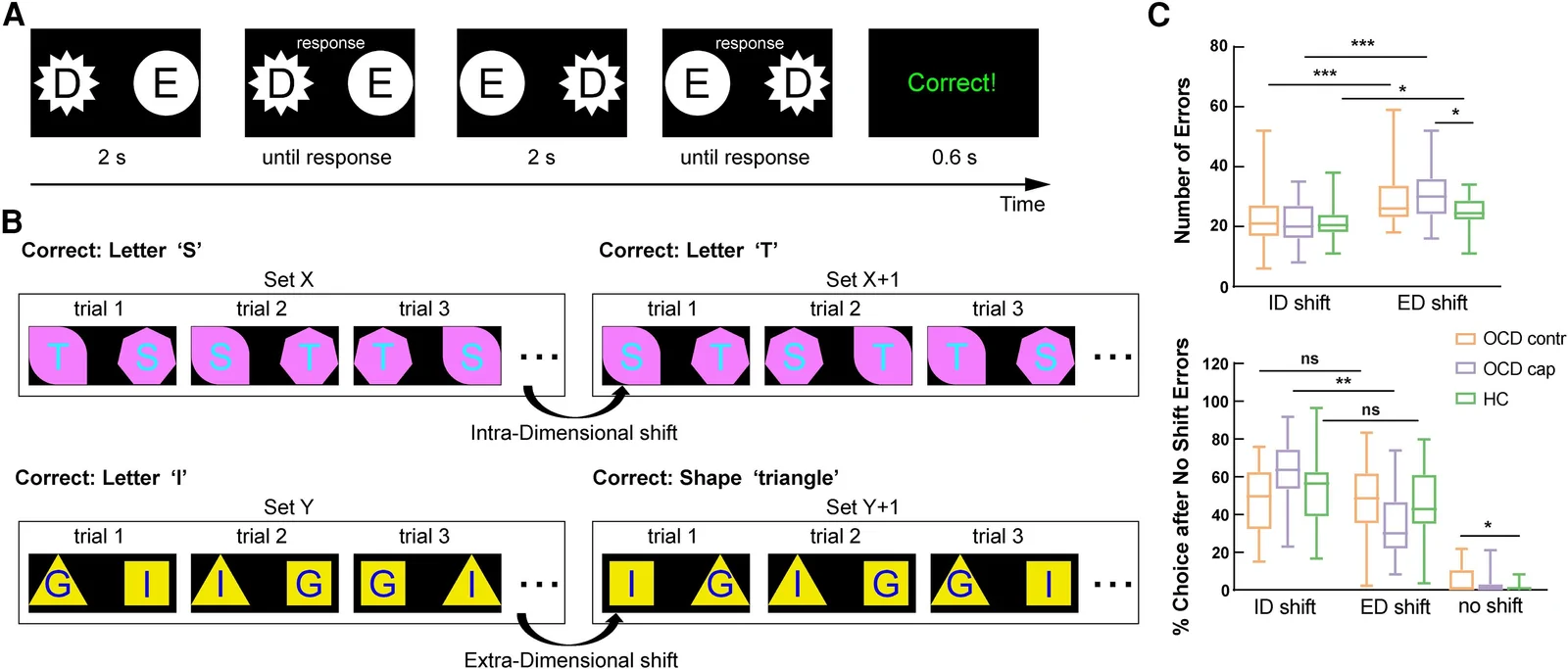
**Between-group differences of set-shifting performances as measured with the Extra-Dimensional Intra-Dimensional (EDID) set-shifting task**. (**A**) Schematic depiction of the EDID task. (**B**) Working procedures of the ID and ED shifts: an ID shift was defined as the shifting to a new target feature from the different dimension (e.g. letter ‘S’ to letter ‘T’); an ED shift was defined as the shifting to a new target feature from a different dimension (e.g. letter ‘I’ to shape ‘triangle’). (**C**) Error rate: significant group by condition interaction driven by greater numbers of errors made during ED shifts in OCD cap group compared with HC. (**D**) Per cent choice: significant group by condition interaction driven by greater per cent no-shift choice made after no-shifts errors in the OCD contr group compared with HC. *P < 0.05, **P < 0.01, ***P < 0.001. OCD contr = OCD control group; OCD cap = OCD capsulotomy group; HC = healthy control; ns = not significant; OCD = obsessive-compulsive disorder.

#### N-back task

We used a conventional block-design N-back task to test working memory (Fig. 2A). The task consisted of three levels: 0-back, 1-back and 2-back. During each block, a series of letters were presented at the centre of the screen. Each letter was displayed for 2.5 s, followed by a 0.5-s inter-stimulus interval. Participants were required to determine whether the current letter matched or did not match the letter ‘X’ in the 0-back condition, or the previous one or two letters in the 1-back and 2-back conditions, respectively. A total of nine blocks were administered, with 15 letters presented in each block. There was a 12-s rest between two blocks. The order of the blocks was randomly determined before the study and counterbalanced across participants.

**Figure 2:**
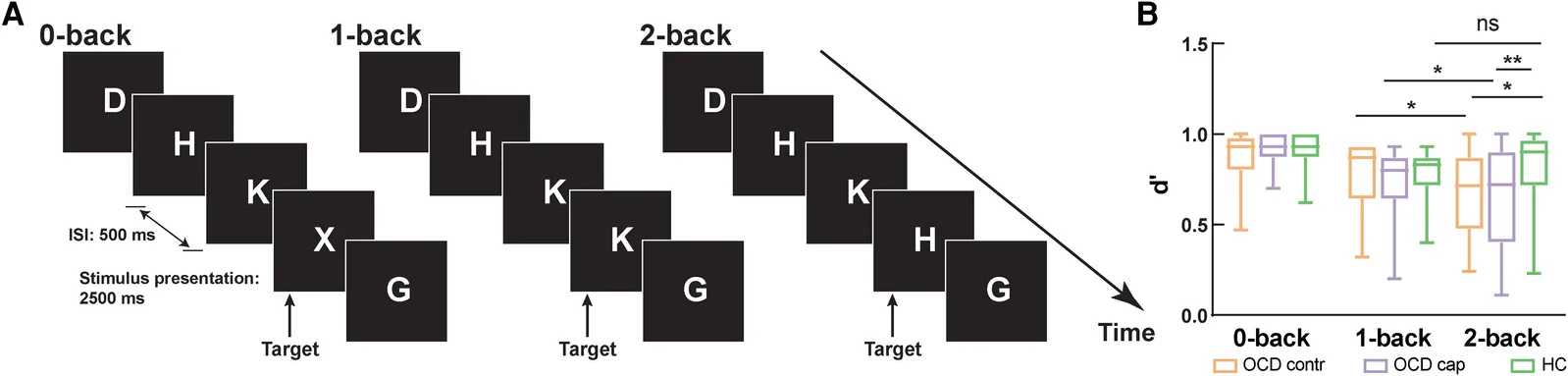
**Between-group differences of working memory performances as measured with the N-back task.** (**A**) Schematic depiction of the N-back task: during the N-back task, participants were asked to determine if the current letter matched a specific target (e.g. ‘X’) in the 0-back condition, or if it matched the letter from one (1-back) or two (2-back) trials back. (**B**) Signal detection measure d’: significant group by condition interaction driven by greater d’ in HC compared with both OCD contr and OCD cap during 2-back conditions, with no differences in between. *P < 0.05, **P < 0.01. OCD contr = OCD control group; OCD cap = OCD capsulotomy group; HC = healthy control; ns = not significant; OCD = obsessive-compulsive disorder.

#### Flanker task

The flanker paradigm (Fig. 3A) was performed to compare behavioural differences in conflict situations but was not a fMRI task in the current cohort. Each trial consisted of two frames: a flanker frame and a target frame. In the flanker frame, a three by five matrix of arrows pointing towards a specific direction was briefly displayed for 50 ms. The middle arrow in the matrix was missing. Following the flanker frame, a target frame was presented, where the middle arrow could either point in the same direction as the surrounding arrows (congruent condition) or in the opposite direction (incongruent condition). The target frame remained visible for 150 ms, and the maximum response period varied randomly between 1100 and 3200 ms to introduce jitter and prevent anticipatory responses. Participants were instructed to respond as quickly as possible by categorizing the target as either ‘congruent’ or ‘incongruent’. Subsequently, a 1000 ms feedback was displayed. In total, there were 200 trials administered throughout the task.

**Figure 3:**
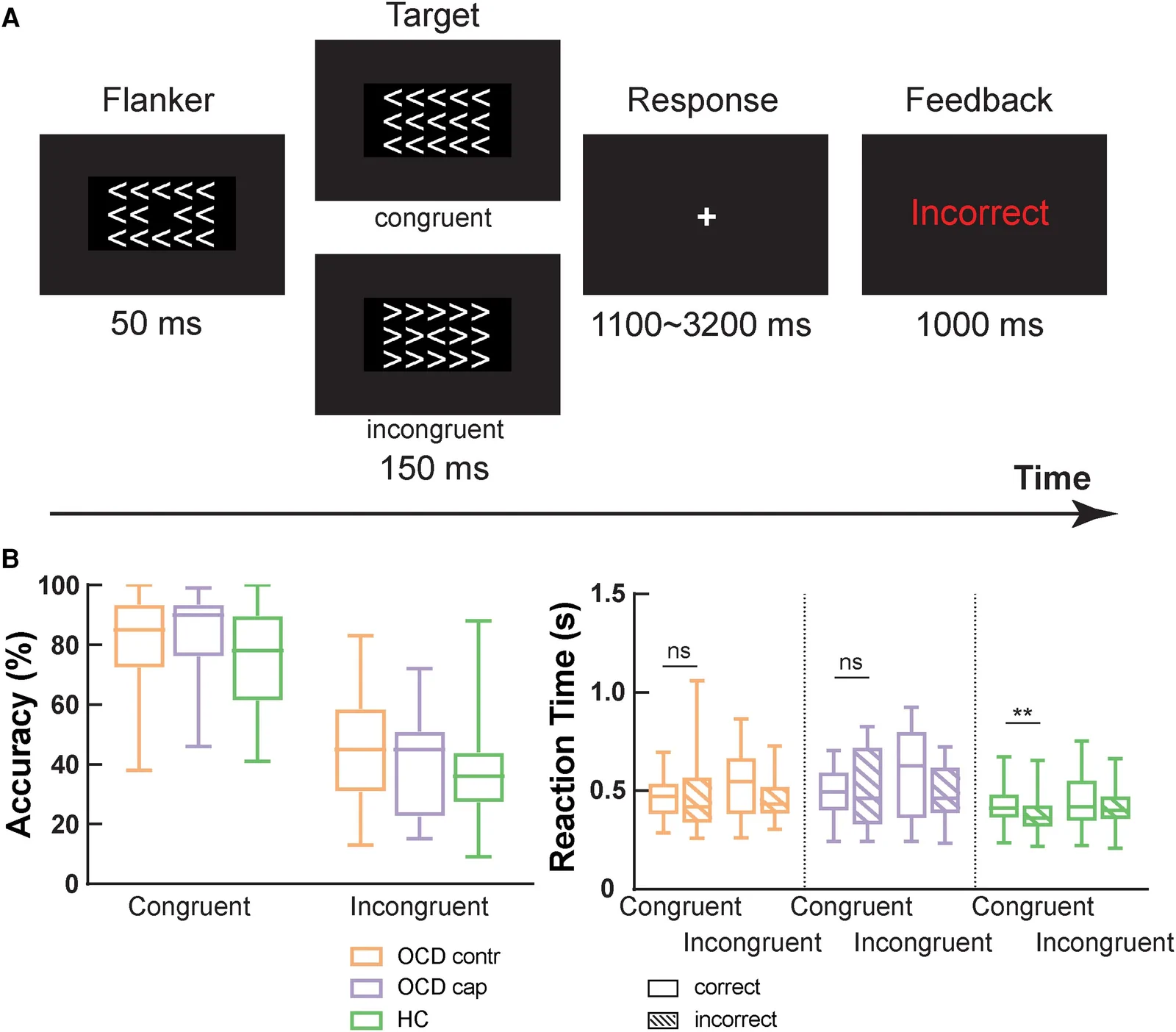
**Between-group differences of action monitoring performances as measured with the flanker task.** (**A**) Schematic depiction of the flanker task. (**B**) *Left*: Significant main effect of congruency was found in the response accuracy, with no group main effect or interaction. *Right*: Significant group by condition interaction driven by longer reaction time for correct trials only in HC group. **P < 0.01. OCD contr = OCD control group; OCD cap = OCD capsulotomy group; HC = healthy control; ns = not significant; OCD = obsessive-compulsive disorder.

### Behaviour data analyses

For the EDID task, we first focused on the average number of errors made within a block for each condition. We also examined responses following a no-shift choice with incorrect feedback, which indicates a behavioural adaptation following an error assessing both error monitoring and post-error adaptation. After a no-shift error, there are three potential choices: ID shift, ED shift and no shift. While the former two shifts are reasonable as this task was deterministic, choosing no shift represents an incorrect response. For the N-back task, we used the d’ as the main dependent variable. The calculation of d’ utilizes signal detection theory as follows: d’ = hit rate – false alarm rate. This approach, frequently employed in working memory assessments, reflects participants’ sensitivity in distinguishing between the two choices (i.e. match/non-match).^32^ In the flanker task, accuracy and reaction time (RT) were calculated for congruent and incongruent trials separately. Post-error RT was also assessed to evaluate error sensitivity and post-error slowing and adaptation. Mixed-effects ANOVAs were used to test group by condition interactions for all task behaviours. *Post hoc* analyses were corrected using Fisher’s least significant difference (LSD) test.

### Statistical analyses

The statistical analyses mainly employed frequentist methods using IBM SPSS statistics 26. Comparisons between conditions were conducted using repeated-measures ANOVAs. Mauchly’s Test of Sphericity was used to test the assumption of sphericity. If sphericity was not assumed, Pillai’s Trace correction was used with multivariate tests. Frequentist *post hoc* analyses were adjusted using the LSD correction for multiple comparisons.

Given our limited sample size, we also reported Bayes factors (BF_10_) calculated using JASP as supplement for both clinical and behavioural testing. The Bayesian framework permits a quantitative evaluation of evidence supporting H1 and H0, offering a distinct advantage in small-sample research. This approach enables a more comprehensive interpretation of the results, avoiding a rigid significant/non-significant dichotomy to better characterize the observed effects.

### General linear model analyses

MRI data acquisition and preprocessing are reported in the Supplementary material and previous publications.^28^ In the EDID task, onsets of the first presentation of acquisition, ED shifts, ID shifts and no shifts (according to participants’ responses) were modelled separately. All presentations of stimuli for the second choice were modelled as one regressor as processing during this frame required replication of the previous choice rather than set-shifting. Button press and feedback were also modelled with the six head movement regressors. We first conducted a whole-brain analysis comparing ED versus ID shift in the HC group to obtain regions-of-interest (ROIs) sensitive to different dimensions of set-shifting. We then extracted beta values from ROIs in all groups after multiple correction [*P* < 0.05 false discovery rate (FDR) cluster corrected, cluster size > 100 voxels] and conducted 2 (shift) × 3 (group) mixed-effect ANOVAs for each ROI to examine the differences. In the N-back task, 0-back, 1-back and 2-back were modelled as blocks separately. The duration of each block was set to 45 s. The six head movement regressors were also included. A 3 (*n*) × 3 (group) repeated-measure ANOVA was used for statistical purposes.

## Results

### Demographics and clinical characteristics

Demographic and clinical characteristics are summarized in Table 1. Depression scores were the highest in OCD contr and lowest in HC, with Bayes factors providing extreme evidence of group differences. Frequentist *post hoc* analyses revealed a significant difference between groups (OCD contr versus OCD cap: *P* = 0.007; OCD contr versus HC: *P* < 0.001; OCD cap versus HC: *P* = 0.001). Bayes factors showed extreme and strong evidence of higher Beck Depression Inventory (BDI) score in OCD contr (BF_10_ = 7.285 × 10^4^) and OCD cap (BF_10_ = 19.525) compared with HC, respectively, with only anecdotal evidence of difference between two OCD groups (BF_10_ = 2.476). No significant differences were found in gender, age or education years across three groups, and Bayesian analyses supported moderate null results only for gender and education years. For the two OCD groups, Yale-Brown Obsessive-Compulsive Scale (Y-BOCS) scores (total, obsessions and compulsions) were higher in OCD contr, supported by both frequentist and Bayesian analyses. Moderate evidence supported no difference in onset age, while only anecdotal evidence supported no difference for disease duration. See Supplementary Table 4 for detailed *post hoc* results for demographic and clinical characteristics of all three groups.

**Table 1:** Demographic and clinical characteristics of the participants

	OCD control (*n* = 30)	OCD capsulotomy (*n* = 23)	HC (*n* = 32)	Bayesian	Frequentist	
				BF_10_	*F*/*t*/χ2	*P*
Gender, female/male	13/17	6/17	14/18	0.247	2.155	0.340
Age, years	30.40 (8.05)	32.83 (6.62)	34.81 (8.79)	0.565	2.360	0.101
Education, years	14.0 (3.53)	13.04 (3.15)	14.31 (3.38)	0.237	1.013	0.368
BDI	22.67 (14.53)	14.57 (11.74)	4.44 (4.33)	27215.500	21.784	<0.001
Y-BOCS total	23.30 (9.17)	12.04 (9.05)	–	1442.071	5.124	<0.001
Obsessions	13.13 (5.39)	6.52 (5.29)	–	555.373	4.762	<0.001
Compulsions	10.17 (6.00)	5.52 (5.12)	–	38.138	3.682	0.005
OCI-R	22.43 (13.60)	13.61 (11.80)	–	2.511	2.338	0.017
Duration, years	10.98 (6.94)	14.37 (6.54)	–	0.418	−0.813	0.096
Onset age, years	18.93 (5.16)	18.62 (4.99)	–	0.323	−0.033	0.829
Medication, ON/OFF	22/8	11/12	–	1.856	3.605	0.058
SSRI, ON/OFF	17/13	10/13	–	0.516	0.906	0.341

Standard deviations are in parentheses = mean (SD). BDI = Beck Depression Inventory; Y-BOCS = Yale-Brown Obsessive-Compulsive Scale (total, obsession, and compulsion scores); OCI-R = Obsessive-Compulsive Inventory-Revised; HC = healthy control; OCD = obsessive-compulsive disorder; SSRI = selective serotonin reuptake inhibitor.

### Behaviour results

#### Extra-Dimensional Intra-Dimensional task

We performed 2 (condition) × 3 (groups) repeated-measure ANOVAs for the number of errors of the EDID task (Fig. 1C, top). To ensure that age and gender did not confound our results, we first conducted a Bayesian analysis using age and gender as covariates. The Bayesian model comparison revealed BF_10_ values of 3.932 × 10^4^, 7.308 × 10^4^ and 1.199 × 10^5^ for interaction models including age, gender and no covariates, respectively, demonstrating the model without age and gender as the best model. Consequently, we did not include age and gender as covariates in the subsequent behavioural analyses of the EDID task. Results showed that the main effect of condition was significant [*F*(1,58) = 46.674, *P* < 0.001, η^2^_p_ = 0.446; BF_10_ = 2.680 × 10^5^], reflecting higher errors made in ED compared with ID shifts. No group difference was revealed by the frequentist analysis [*F*(2,58) = 1.066, *P* = 0.351, η^2^_p_ = 0.035], which was anecdotally supported by the Bayesian analysis (BF_10_ = 0.353). A significant interaction was found [*F*(2,58) = 3.521, *P* = 0.036, η^2^_p_ = 0.446; BF_10_ = 1.516], driven by more errors made in OCD cap compared with HC in the ED shift (*P* = 0.025; BF_10_ = 4.562) but not in the ID shift (*P* = 0.863; BF_10_ = 0.324). For all three groups, less errors were made in ID compared with ED shifts (OCD contr: *P* < 0.001, BF_10_ = 29.30; OCD cap: *P* < 0.001, BF_10_ = 91.49; HC: *P* = 0.023, BF_10_ = 4.693).

A critical measure is whether one stays or switches after an error. We examined responses following a no-shift choice with incorrect feedback (Fig. 1C, bottom). After a no-shift error, there were three potential choices: ID shift, ED shift and no shift. While the former two shifts were reasonable, choosing no shift represented an incorrect/inflexible response. By using a 3 (shifts) × 3 (groups) repeated-measure ANOVA, we observed a significant interaction effect [*F*(4,116) = 2.825, *P* = 0.028, η^2^_p_ = 0.089; BF_10_ = 6.565]. In *post hoc* analyses, a significantly larger proportion of no-shift choices after no-shift errors was found in OCD contr compared with HC (*P* = 0.020; BF_10_ = 3.106). No differences were found between OCD cap versus HC (*P* = 0.322) or OCD contr versus OCD cap (*P* = 0.265), with anecdotal evidence supporting null results of the two comparisons (BF_10_ = 0.631 and 0.446, respectively). These findings indicated that OCD patients were more likely to stay and make suboptimal inflexible choice behaviours following an error, which may be remediated to some extent after capsulotomy. OCD cap patients also showed a greater likelihood of making ID shifts relative to ED shifts after no-shift errors (*P* = 0.006; BF_10_ = 5.395) while this pattern was not exhibited in the other two groups (OCD contr: *P* = 0.950, BF_10_ = 0.223; HC: *P* = 0.356, BF_10_ = 0.308).

#### N-back task

For the N-back task, we focused on the index of d’ using 3 (conditions) × 3 (groups) repeated-measure ANOVAs (Fig. 2B). The Bayesian model including covariates showed that the BF_10_ of interaction models with age or gender were 2.613 × 10^9^ and 2.085 × 10^9^, respectively, while the BF_10_ of the interaction model without age or gender was 4.855 × 10^9^, indicating that adding age or gender may worsen the model fitting. Thus, we did not include them in the following analyses. Main effect of d’ [*F*(2,74) = 36.475, *P* < 0.001, η^2^_p_ = 0.496; BF_10_ = 1.84 × 10^9^] and interaction [*F*(4,150) = 3.077, *P* = 0.018, η^2^_p_ = 0.076; BF_10_ = 5.768] were observed, while the group difference was insignificant indicated by the frequentist analysis [*F*(2,75) = 2.120, *P* = 0.127, η^2^_p_ = 0.053] with anecdotal evidence supporting no difference according to the Bayesian analysis (BF_10_ = 0.549). In the *post hoc* analyses, lower d’ was only found in the 2-back condition for OCD contr (*P* = 0.027; BF_10_ = 3.783) and OCD cap (*P* = 0.006; BF_10_ = 5.381) compared with HC, with no difference between OCD contr and OCD cap (*P* = 0.537; BF_10_ = 0.327). Moreover, OCD contr (*P* = 0.024; BF_10_ = 1.294) and OCD cap (*P* = 0.028; BF_10_ = 1.114) showed lower d’ in 2-back compared with 1-back, which was not shown in HC (*P* = 0.244; BF_10_ = 0.604).

#### Flanker task

In the flanker task, accuracy was firstly analysed by a 2 (conditions) × 3 (groups) repeated-measure ANOVA (Fig. 3B, left). Again, the Bayesian model with age or gender as covariates revealed a BF_10_ = 2.128 × 10^22^ and 1.442 × 10^22^, respectively, and BF_10_ of the model without age or gender was 5.823 × 10^22^, indicating the best model without age or gender. Therefore, the following analyses did not include covariates. The main effect of congruency was significant [*F*(1,68) = 180.091, *P* < 0.001, η^2^_p_ = 0.726; BF_10_ = 1.485 × 10^23^], while no differences were found for the group effect [*F*(2,68) = 1.666, *P* = 0.197, η^2^_p_ = 0.047; BF_10_ = 0.421] or interaction [*F*(2,68) = 0.803, *P* = 0.452, η^2^_p_ = 0.023; BF_10_ = 0.205].

We further divided reaction times into correct and incorrect trials for congruent and incongruent conditions and ran a 2 (congruency) × 2 (correctness) × 3 (group) repeated-measure ANOVA (Fig. 3B, right). Main effects of condition [*F*(1,67) = 21.108, *P* < 0.001, η^2^_p_ = 0.240; BF_10_ = 24.809] and correctness [*F*(1,67) = 37.419, *P* < 0.001, η^2^_p_ = 0.358; BF_10_ = 5.911 × 10^3^], as well as their interaction [*F*(1,67) = 9.330, *P* = 0.003, η^2^_p_ = 0.122; BF_10_ = 19.236] were significant and supported H1-favoured hypothesis by Bayesian analyses, indicating a robust flanker effect. The three-way interaction was also significant [*F*(2,67) = 3.511, *P* = 0.035, η^2^_p_ = 0.095; BF_10_ = 15.423]. *Post hoc* analyses showed that in the congruent condition, HC exhibited lower RTs for incorrect trials (*P* = 0.007; BF_10_ = 5.209 × 10^4^), while such a difference was not found in OCD contr (*P* = 0.626; BF_10_ = 0.225) and OCD cap (*P* = 0.884; BF_10_ = 0.280). On the other hand, a group effect was also found [*F*(2,67) = 4.250, *P* = 0.018, η^2^_p_ = 0.113; BF_10_ = 3.139] indicated by generally longer RTs for OCD contr (*P* = 0.043; BF_10_ = 382.047) and OCD cap (*P* = 0.010; BF_10_ = 1.991 × 10^4^) compared with HC. These findings suggest that in HC, incorrect answers were associated with faster and more impulsive decision-making. In contrast, in OCD patients, particularly when the task demands were low, they tended to invest more effort and time, even in making incorrect responses.

### Functional MRI results

#### Extra-Dimensional Intra-Dimensional task

Similar to the way we conducted our previous imaging analysis in this cohort,^28^ we first conducted a whole-brain analysis to identify ROIs implicated in different dimensions of set-shifting in HC by comparing ED versus ID activity (*P* < 0.05 FDR cluster corrected, cluster size > 100 voxels; Supplementary Table 5). Seven clusters were extracted and we then conducted a 2 (shifts) × 3 (groups) repeated-measures ANOVA for each cluster (Fig. 4). Significant interactions were found in cluster 1 [*F*(2,58) = 3.439, *P* = 0.039, η_p_^2^ = 0.106], cluster 2 [*F*(2,58) = 5.348, *P* = 0.007, η_p_^2^ = 0.156], cluster 3 [*F*(2,58) = 3.810, *P* = 0.028, η_p_^2^ = 0.116] and cluster 4 [*F*(2,58 = 3.382, *P* = 0.041, η_p_^2^ = 0.104], with *post hoc* analyses indicating greater brain activity during ED versus ID shifting in the HC group, including the bilateral pre-supplementary motor area (pre-SMA) and dorsolateral prefrontal cortex (dlPFC). Specifically with ED shift, OCD cap patients showed lower activity in bilateral pre-SMA compared with HC (*P* = 0.021) but not ID shifts, which may underlie the increased error rates observed in OCD cap specifically during ED shifts (Fig. 1C). For cluster 5 [*F*(2,58) = 1.753, *P* = 0.182, η_p_^2^ = 0.057], cluster 6 [*F*(2,58) = 2.001, *P* = 0.144, η_p_^2^ = 0.065] and cluster 7 [*F*(2,58) = 1.197, *P* = 0.309, η_p_^2^ = 0.040] no interactions were revealed, reflecting relatively intact function in parietal regions in OCD.

**Figure 4:**
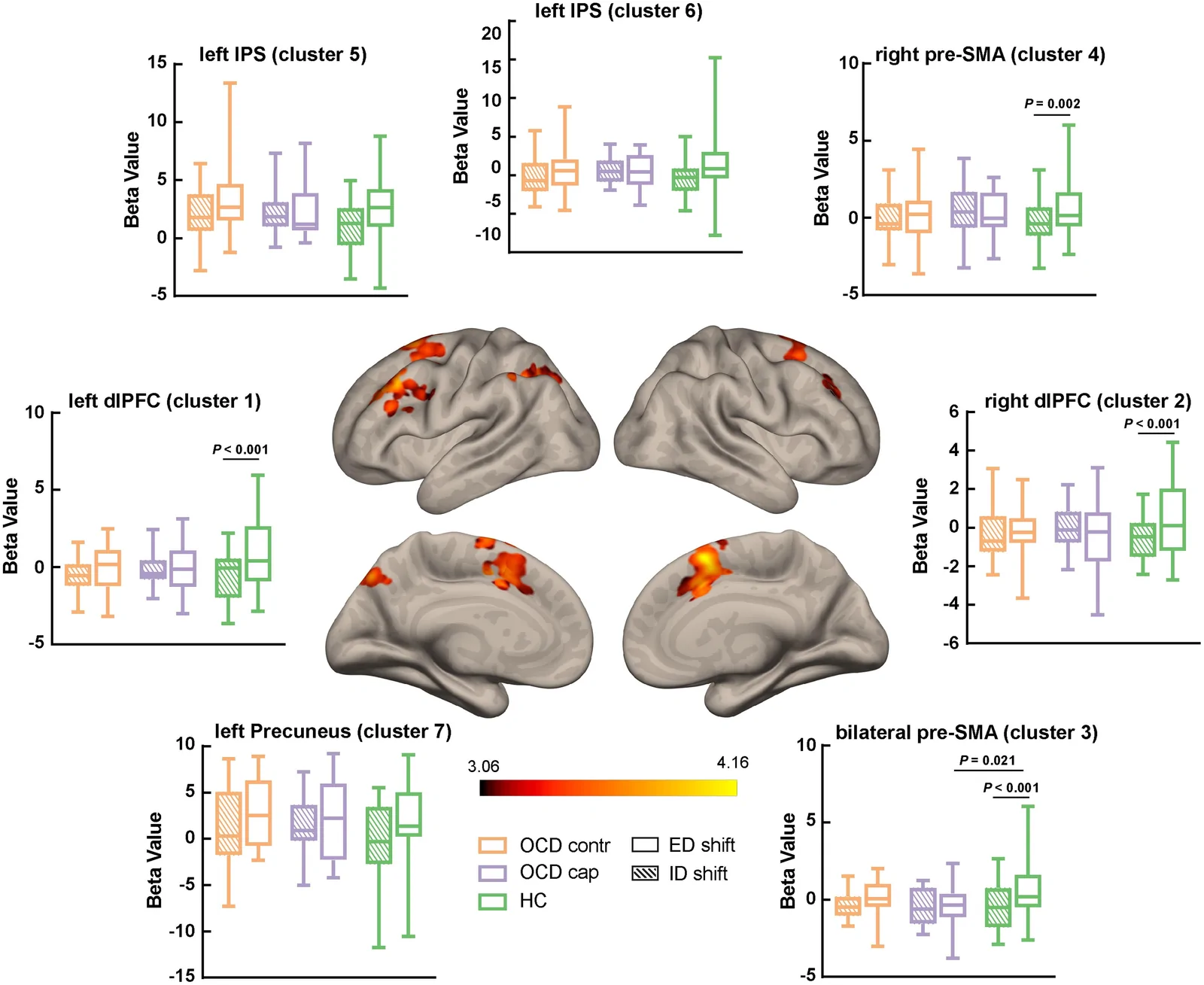
**Regional brain activity results underlying functional response to Extra-Dimensional Intra-Dimensional (EDID) set-shifting task**. Brain areas, including the pre-supplementary motor cortex (pre-SMA) and left dorsolateral middle frontal cortex (dlPFC), have shown higher ED shift-induced activity in the HC group compared with OCD cap and OCD contr. In HC participants, higher regional activity during ED-shift in contrast with ID-shift were shown in all four clusters of significance, including the pre-SMA and bilateral dlPFC. Bilateral pre-SMA showed lower activity between HC and OCD cap during ED-shift. OCD contr = OCD control group; OCD cap = OCD capsulotomy group; HC = healthy control; ID = intra-dimensional shift; ED = extra-dimensional shift; ns = not significant; OCD = obsessive-compulsive disorder.

#### N-back task

In the N-back task, we conducted a 3 (loads) × 3 (groups) repeated-measure ANOVA. No voxels survived interaction after multiple comparison. The main effect of memory load was significant (*P* < 0.05 FDR cluster corrected) indicating a robust working memory effect in the frontoparietal network (Supplementary Fig. 1).

## Discussion

We highlight the effects of anterior capsulotomy in OCD on behavioural flexibility following errors in the EDID task. The shift appears to be associated with greater ID relative to ED flexibility with the lower ED post-error flexibility possibly also indicating greater ED shift errors. OCD patients showed less neural differentiation between ED versus ID in lateral and mesial prefrontal regions relative to HC. Capsulotomy was associated with lower pre-SMA activity to ED shifts compared with HC indicating impairments in neural reactivity to ED shifting. In contrast, OCD patients had enduring impaired working memory particularly for difficult contexts and slower responding during incorrect answers in response to conflict, both showed limited influence caused by capsulotomy. Critically, capsulotomy improved OCD symptom severity along with quality of life and disability^7^ suggesting the improvement in flexibility post-error may be state related to symptom improvement whereas the enduring impairments in working memory and conflict response may reflect trait markers or risk factors for the disease unrelated to symptom change and treatment response.

### Cognitive inflexibility and error monitoring

Cognitive flexibility or attentional set-shifting is impaired in OCD across numerous paradigms, ranging from traditional tasks such as the Wisconsin Card Sort Task (WCST)^33,34^ and the Trail-Making Task (TMT)^17,35^ to the EDID task^13^ which dissociates ED from ID shifting.^13^ We found significantly more ED errors in the OCD cap group compared with HC. In OCD controls we observed a numerical but not statistical difference compared with HC suggesting this lack of difference to be related to sample size similar to other observations in the literature^36,37^ rather than an effect of capsulotomy per se. Moreover, OCD capsulotomy patients appeared to show lower pre-SMA activity to ED relative to HC. Thus, OCD capsulotomy targeting the ALIC does not improve ED shifting in OCD, and indeed might be associated with greater ED errors supported by greater deactivation in the pre-SMA relative to HC. This impairment in ED shifting in OCD capsulotomy versus HC, which was not observed in OCD controls versus HC, has several potential interpretations. Critically, we emphasize that capsulotomy appears to improve OCD symptoms without necessarily being mediated through improving this specific form of cognitive flexibility. In our previous publication we also reported trend group differences (which could not survive multiple correction) showing a greater number of incorrect responses across ID/ED conditions in both capsulotomy patients and OCD controls compared with HC.^7^ This result aligns with our current findings as both findings potentially suggest that capsulotomy does not appear to improve either ED or ID shifting. An alternate explanation is that capsulotomy might have a negative effect on impairing ED shifting and decreasing pre-SMA activity. This is a plausible explanation although we show little supporting evidence. ED shifting impairments are a robust and reproducible finding in OCD (effect size medium-large) and further show a trait-level effect with impairments observed in unaffected family members.^13,36,37^ As such, that we did not observe ED shift impairments in the OCD controls versus HC was unexpected, and along with the lack of ED shift differences between OCD cap and OCD contr, markedly limits our interpretation of the ED impairments observed between OCD cap and HC. Given the small sample size, we believe the unexpected lack of ED impairment in the OCD controls versus HC is much more likely related to a sample size issue consistent with lack of an effect of capsulotomy on ED impairments rather than a negative effect of capsulotomy in the OCD cap group. Further longitudinal studies are indicated with larger sample sizes to fully address the effects of capsulotomy on ED shifting.

A DBS study has shown that targeting the ventral capsule/ventral striatal (VC/VS), which is part of the ALIC, improves depressive symptoms but not cognitive flexibility,^38^ indicating different neuromodulation mechanisms underlying ablation and DBS. Meanwhile, targeting the anteromedial STN (amSTN) behaviourally improving ED shifting,^38^ implying that OCD patients with marked impairments in ED shifting may potentially be benefited from DBS of the amSTN. However, that OCD symptoms improve post-capsulotomy without any clear change in ED shifting constrains this as a potential clinical predictor at least in the context of OCD capsulotomy.

Critically, in this study, OCD controls were less likely to shift following an error compared with HC, while no such impairment was found in OCD caps. This deficit specific to the response after errors is consistent with hyperactive error monitoring, a process that can be described in OCD as monitoring thoughts or actions more carefully to avoid losing control or committing errors. It has been proposed that error monitoring is intrinsically associated with cognitive inflexibility, which prevents OCD patients from shifting cognitive strategies and adapting to new rules when situation demands.^39^ However, our findings suggest a potential effect of capsulotomy not on behavioural inflexibility but on error monitoring implicating the mesial prefrontal cortex with subsequent greater flexibility post-errors.

### Working memory

Both OCD groups show working memory impairment and poorer performance in higher loads,^40,41^ while capsulotomy probably has no influence in this function (but only supported by anecdotal evidence according to the Bayesian analysis). This aligns with our own findings in the same cohort showing no working memory changes post-capsulotomy using the Cambridge Neuropsychological Test Automated Battery (CANTAB) spatial working memory task,^7^ as well as with other studies reviewed in the systematic analysis.^27,42^ Improved performance in executive tasks, however, has been observed with other forms of brain modulation.^43^ Despite inconsistencies,^44,45^ transcranial magnetic stimulation targeting bilateral dlPFC and high-definition transcranial alternating-current stimulation (tACS) targeting the left prefrontal cortex and the left temporal cortex^46^ have both been reported to improve working memory in OCD (e.g. digit span backward).^47^

### Flanker task: cautious responding during errors

The flanker task assesses basic attentional processing with congruent stimuli, while examining action monitoring and response inhibition with prepotent responses to incongruent stimuli. We found longer response latency among OCD participants in response to incorrect trials during congruent conditions, suggesting faster and more impulsive decision-making for incorrect answers in HC, whereas OCD patients invested more effort and time, even when their responses were incorrect. This result aligns with our previous findings from the same cohort using a card gambling task, which demonstrated increased caution, worse performance and greater evidence accumulation.^28^ OCD patients are generally slower in making decisions compared with controls and tend to gather more evidence during the process, indicating cautiousness in decision-making under certain circumstances.^28,48^ This may be driven potentially by overactive action monitoring, causing patients to fear ambiguous situations where errors are likely to occur.^49^ This greater caution during conflict errors and during uncertainty in OCD appears unaffected by capsulotomy suggesting a potential trait-related marker of OCD rather than state dependent.

### OCD and capsulotomy effects on brain functional activity

Set-shifting implicates the lateral frontoparietal and mediofrontal networks in supporting executive function and cognitive flexibility.^50-52^ In the current study, we observed greater right dlPFC and bilateral pre-SMA during the set-shifting process in HC, with OCD cap demonstrating significant lower activity in pre-SMA during ED shifting compared with HC. These findings align with decreased activity in OCD patients in these same regions during set-shifting tasks.^50,52^ Despite behavioural changes in post-error flexibility in capsulotomy patients compared with OCD contr, no change in the corresponding ROI analyses was found. There are several potential explanations for this dissociation which might include compensatory recruitment of neural substrates outside the measured ROIs, methodological issues with sensitivity of the specific task for post-error adaptation and limited statistical power given the small sample size. Our findings suggest that OCD postoperative cognitive changes within specific domains might have relevance to the anterior–posterior ventral–dorsal tract organization arranged topographically within the striatum.^4^ We show that capsulotomy may be associated with impaired activity during ED shifting in the mesial prefrontal pre-SMA but we did not observe any specific effects of capsulotomy on lateral prefrontal processes typically involved in executive processing. Likewise, we found no significant between-group differences in brain activity for the N-back task, except for a significant main effect of memory load (1-, 2- and 3-back) in the lateral prefrontal area, indicating a robust working memory effect. Our previous studies in this same cohort showed downregulation of hyperactive rostral dorsal cingulate regions with aversive expectation and conditioning following capsulotomy.^7,28^ Thus, one plausible explanation is that capsulotomy has a greater effect on normalizing aberrant mesial prefrontal activity rather than lateral prefrontal activity, and that this effect may have a greater effect on improving treatment outcomes. Capsulotomy appears to have less influence on the lateral prefrontal executive processes as compared with mesial prefrontal aversive processing.^7^ Further studies involving measures such as inhibition that also implicate the ventrolateral prefrontal cortex or using tractography are indicated to further elucidate this hypothesis. Furthermore, our observation of possibly greater ED errors and decreased pre-SMA activity during ED shift might suggest limiting the dorsal extent of capsulotomy might improve ED shifting. However, given the improvement in OCD Y-BOCS score post-capsulotomy, how this might link to or impact OCD symptoms remains unclear.

### Limitations

This study is not without limitations. Firstly, our study is quite small in sample size and included fMRI tasks examining the changes of executive functions after surgery given time constraints. As this study was part of a larger study in which we investigated other OCD-relevant cognitive mechanisms known to map across prefrontal regions connected to the ALIC tracts targeted in capsulotomy, we were only able to selected three executive paradigms. We assessed other executive domains (including inhibition and planning functions) using behavioural tasks and have reported the results in a previous publication.^7^ Briefly, executive planning revealed no group difference using the Stockings of Cambridge paradigm following multiple comparisons; trend group differences (*P* = 0.08) were found in response inhibition (Stop signal task) in which post-capsulotomy subjects showed greater inhibitory impairments relative to OCD controls with the addition of Y-BOCS scores as a covariate. Further functional task-based MRI studies addressing the effects of inhibition may be indicated given our trend-level behavioural findings.

Secondly, the impact of capsulotomy on executive function, the ‘trait versus state’ hypothesis in particular, while mechanistically plausible, should ideally be examined by comparing within-subject pre- and post-surgery performance on executive paradigms. As the study was designed as a parallel study, we cannot rule out neurocognitive differences in the executive domain between OCD contr and OCD cap before the surgical intervention. Further research including longitudinal or familial comparison to clarify this putative ‘trait versus state’ effect on executive functions following neuroablation is needed. It is also noteworthy that, beyond investigating how capsulotomy reshapes executive function, another key objective of this study was to explore how capsulotomy alters brain executive functions in ways that distinguish treated patients from non-refractory OCD patients. Future longitudinal studies incorporating pre- and post-surgical assessments on behaviour and function will be critical for demonstrating executive-circuit remodelling following capsulotomy.

## Conclusion

We highlight the remodelling effect of capsulotomy on potentially improving error monitoring processes implicating particularly mesial prefrontal regions in OCD. In contrast, lateral prefrontal cortical activity implicated in working memory and conflict appears unaffected and may relate to stable trait markers in OCD. These findings have relevance in guiding more accurate selection of neuromodulation targets and techniques, highlighting the need for larger-scale studies to predictive biomarkers for the safety profile of capsulotomy.

## Supplementary Material

awag132_Supplementary_Data

## Data Availability

All data reported in this paper will be shared by the corresponding author upon reasonable request.
