# Supplementary material for: Dissociating executive prefrontal substrates following capsulotomy in obsessive-compulsive disorder
Source: Brain. 2026 Apr 16;149(11):3837–47. doi: 10.1093/brain/awag132 (PMC13730558; doi:10.1093/brain/awag132)
Supplement: Supplementary file 1 [file awag132_supplementary_data.pdf]

Supplementary information

SI Materials and Methods

Participants and patient selection

The inclusion and exclusion criteria for patients with obsessive-compulsive disorder (OCD) who underwent capsulotomy have been extensively reported.[1-3] They were considered to be refractory and selected to undergo anterior capsulotomy if they met the following criteria: (i) a primary OCD diagnosis according to the Structured Clinical Interview for DSM-V; (ii) a score on the Yale-Brown Obsessive Compulsive Scale (Y-BOCS) of more than 25 or one subscale score of more than 15; (iii) more than 18 years old, with a course of illness at least 5 years; (iv) non-responsive to or were unable to tolerate at least two trials of selective serotonin reuptake inhibitors of adequate duration and dose; and failed to tolerate augmentation with an atypical antipsychotic; and failed or were unable to tolerate psychotherapy/cognitive behavioural therapy (defined as continuous treatment for more than six months). The exclusion criteria were as follows: (i) Schizophrenic disorder; bipolar disorder; substance abuse or dependence (except for dependence on nicotine), as assessed with the use of the Mini-International Neuropsychiatric Interview (MINI 6.0.0); (ii) a current severe major depressive episode, determined according to DSM-V criteria (as assessed with the use of the MINI 6.0.0). OCD control patients should have a primary diagnosis of OCD according to DSM-V and age over 18. The exclusion criteria are the same with the OCD capsulotomy patients. Healthy controls needed to be (i) over the age of 18; (ii) No obvious physical or mental disorders; and (iii) a Beck Depression Inventory Second Edition (BDI-II) score less than 19.

MRI data acquisition and preprocessing

MRI data were collected using a Siemens 3T Prisma scanner and a 64-channel RF coil at Fudan University. Whole-brain T1-weighted imaging was conducted with an MPRAGE sequence: TR 3000 ms, TE 2.56 ms, flip angle 7°, matrix size 320 × 320, Field-of-view 256 mm, voxel size 0.8 mm × 0.8 mm, slice thickness 0.8 mm.Task-based functional images were acquired with an Echo-Planar Imaging (EPI) sequence: TR 2000 ms, TE 30 ms, flip angle 90°, matrix size 96 × 96 with parallel imaging acceleration (GRAPPA) 2, field-of-view 192 mm, voxel size 2 mm × 2 mm, slice thickness 3 mm with a distance factor of 25%. The number of slices was 33. Phase encoding direction was anterior to posterior.

SI Tables

Table S1. Demographic and Clinical Characteristics of the Participants for EDID Task

	OCD contr (n = 22)	OCD cap (n = 15)	HC (n = 24)	Bayesian	Frequentist	
				BF$_{10}$	F/t/χ^2	p
Gender (female/male)	10/12	3/12	11/13	0.545	3.120	.210
Age	29.60 (6.71)	31.78(6.57)	33.83 (8.35)	0.565	1.893	.160
Education (year)	14.64(3.26)	13.53(3.04)	14.83(3.43)	0.237	.785	.461
BDI	22.50(13.77)	12.53(11.12)	4.17(4.03)	27215.5	18.396	< .001
Y-BOCS total	24.82(7.55)	10.93(8.84)	-	1442.071	5.124	< .001
Obsessions	13.55(4.64)	5.80(5.17)	-	555.373	4.762	< .001
Compulsions	11.27(5.01)	5.13(4.94)	-	38.138	3.682	< .001
OCI-R	22.32(13.16)	12.27(12.34)	-	2.511	2.338	.025
Duration	10.70(5.98)	12.27(5.35)	-	0.418	-0.813	.422
Onset age	18.68(4.06)	18.73(5.48)	-	0.323	-0.033	.974

Abbreviations: BDI: Beck Depression Inventory; Y-BOCS: Yale-Brown Obsessive-Compulsive Scale (total, obsession, and compulsion scores); OCI-R: Obsessive-Compulsive Inventory-Revised; OCD contr: OCD control; OCD cap: OCD capsulotomy; HC: healthy control. Standard deviations are in parentheses: mean (SD).

Table S2. Demographic and Clinical Characteristics of the Participants for Working Memory Task

	OCD contr (n = 26)	OCD cap (n = 23)	HC (n =29)	Bayesian	Frequentist	
				BF$_{10}$	F/t/χ^2	p
Gender (female/male)	10/16	6/17	12/17	0.178	1.415	.493
Age	30.50(8.26)	32.74(6.70)	34.24(8.67)	0.361	1.509	.228
Education (year)	14.00(3.78)	13.04(3.15)	14.76(3.00)	0.414	1.708	.188
BDI	22.69(15.20)	14.57(11.74)	4.83(4.36)	31352.799	17.669	<.001
Y-BOCS total	22.31(8.83)	12.04(9.05)	-	114.623	4.014	<.001
Obsessions	12.58(5.52)	6.52(5.29)	-	86.018	3.907	<.001
Compulsions	9.73(5.56)	5.52(5.12)	-	5.460	2.743	.009
OCI-R	23.08(13.88)	13.61(11.80)	-	3.749	2.554	.014
Duration	11.40(7.30)	14.61(6.64)	-	0.394	-0.889	.145
Onset age	19.19(5.46)	18.85(5.01)	-	0.302	0.367	.828

Abbreviations: BDI: Beck Depression Inventory; Y-BOCS: Yale-Brown Obsessive-Compulsive Scale (total, obsession, and compulsion scores); OCI-R: Obsessive-Compulsive Inventory-Revised; OCD contr: OCD control; OCD cap: OCD capsulotomy; HC: healthy control. Standard deviations are in parentheses: mean (SD).

Table S3. Demographic and Clinical Characteristics of the Participants for Flanker Task

	OCD contr (n =26)	OCD cap (n =13)	HC (n =32)	Bayesian	statistics	
				BF$_{10}$	F/t/χ^2	p
Gender (female/male)	13/13	1/12	14/18	4.181	6.949	.031
Age	29.08(7.62)	33.38(6.58)	34.81(8.79)	2.371	3.781	.028
Education (year)	14.73(3.00)	12.77(3.52)	14.31(3.38)	0.388	1.602	.209
BDI	19.46(12.07)	13.92(13.27)	4.44(4.33)	33965.968	17.797	<.001
Y-BOCS total	23.00(9.63)	11.38(9.90)	-	26.936	3.517	.001
Obsessions	12.69(5.66)	5.92(5.30)	-	32.037	3.593	<.001
Compulsions	10.31(6.04)	5.46(5.03)	-	3.278	2.488	.017
OCI-R	22.92(14.36)	11.15(9.81)	-	4.428	2.653	.012
Duration	10.17(6.37)	13.00(6.15)	-	0.638	-1.321	.195

Onset age	18.96(5.17)	19.38(5.71)	-	0.333	-0.233	.817

Abbreviations: BDI: Beck Depression Inventory; Y-BOCS: Yale-Brown Obsessive-Compulsive Scale (total, obsession, and compulsion scores); OCI-R: Obsessive-Compulsive Inventory-Revised; OCD contr: OCD control; OCD cap: OCD capsulotomy; HC: healthy control. Standard deviations are in parentheses: mean (SD).

Table S4. Post-hoc results for Demographic and Clinical Characteristics involving participants from all three groups

		**Bayesian (BF_{10})**			**Frequentist (*p*-value)**		
		**OCD contr v.s. OCD cap**	**OCD contr v.s. HC**	**OCD cap v.s. HC**	**OCD contr v.s. OCD cap**	**OCD contr v.s. HC**	**OCD cap v.s. HC**
EDID	Gender (female/male)	1.250	0.352	1.328	.111	.979	.102
	Age	0.469	1.198	0.410	.651	.136	.679
	Education (year)	0.490	0.297	0.558	.577	.977	.456
	BDI	2.476	72845.380	19.525	.014	<.001	.042
WM	Gender (female/male)	0.482	0.326	0.600	.357	.825	.250
	Age	0.441	0.816	0.339	.593	.200	.780
	Education (year)	0.414	0.362	1.415	.576	.676	.161
	BDI	1.594	76768.642	167.359	.034	<.001	.007
Flanker	Gender (female/male)	11.81	.355	5.603	.009	.635	.020
	Age	1.031	4.310	0.355	.260	.023	.851
	Education (year)	1.147	0.296	0.661	.189	.879	.329
	BDI	0.629	498040.331	39.933	.217	<.001	.011

Abbreviations: BDI: Beck Depression Inventory; OCD contr: OCD control; OCD cap: OCD capsulotomy; HC: healthy control. Gender is analysed using chi-square. Age, education and BDI are analysed using one-way ANOVA and p-values are adjusted by Tukey.

Table S5. Brain Regions Underlying Significant Differences in Bold Activities During ED versus ID Conditions.

Cluster	Region	Hemi	Peak MNI coordinate			T	Cluster size
			x	y	z		
Cluster 1	dlPFC/MFG	L	-42	22	38	4.29	500
Cluster 2	dlPFC/MFG	R	28	30	26	3.61	136
Cluster 3	SMA/SFG, MCC	R	8	16	58	4.44	1568
Cluster 4	SMA	R	22	16	56	3.79	106
Cluster 5	IPS	L	-26	-66	40	4.12	358
Cluster 6	IPS	L	-50	-40	42	3.88	116
Cluster 7	Precuneus	L	-8	-78	46	3.96	163

Abbreviations: L-left; R-right; MNI-Montreal Neurological Institute. SMA-supplementary motor area; SFG-superior frontal gyrus; MCC-middle cingulate cortex; dlPFC-dorsolateral prefrontal cortex; MFG-middle frontal gyrus; IPS- inferior parietal sulcus; hemi-hemisphere. $p<.05$ FDR cluster corrected, cluster size > 100 voxels.

SI Figures

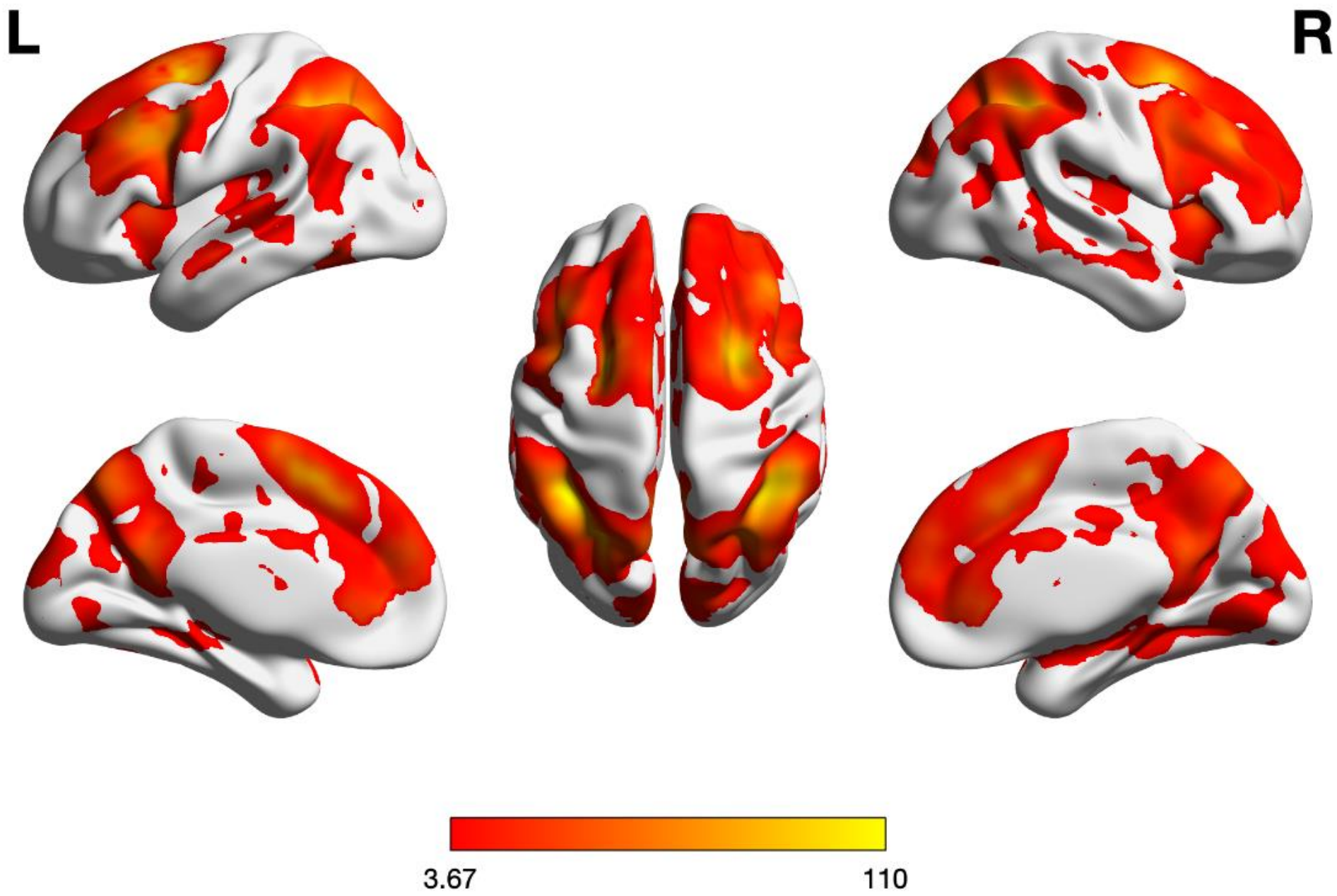

Figure S1. **Functional magnetic resonance imaging results of the n-back task.** Activations detected with a whole-brain analysis involving an F-test for the main effect of working memory load. Clusters displayed are FDR corrected with p<.05 and cluster size > 100. The bar on the right represents the t-statistic.

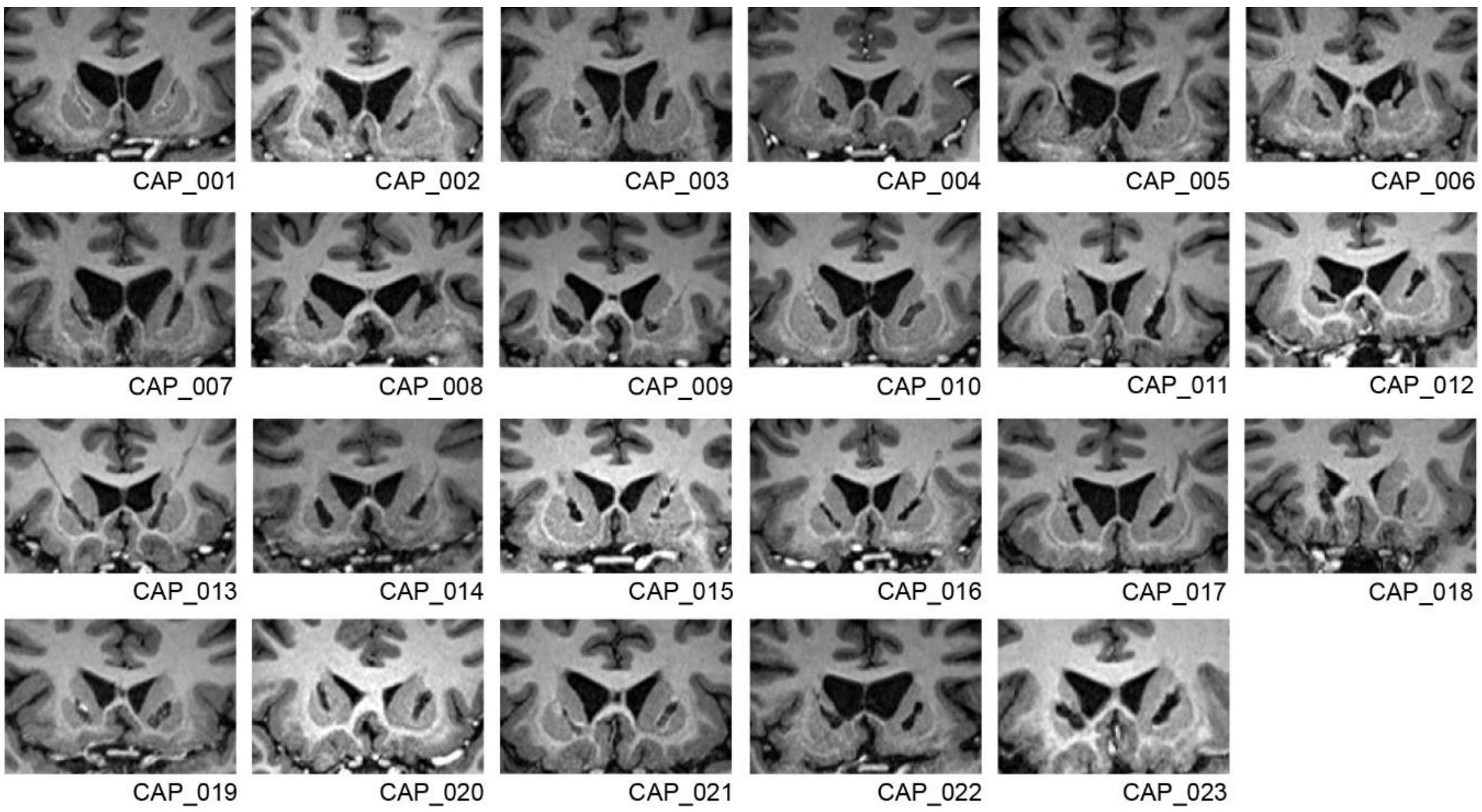

Figure S2. **Capsulotomy lesion extent/size.** The lesion locations for each patient (n=23) are shown in high-resolution T1-weighted images (**Figure S2**). Mean lesion size was 1570.4 mm^3

(standard deviation = 769.2; range 701.0 – 3912.0 mm^3). For more detailed information on the surgical procedure please refer to our previously published work. [3]

References

1. Jung HH, Kim SJ, Roh D, et al. Bilateral thermal capsulotomy with MR-guided focused ultrasound for patients with treatment-refractory obsessive-compulsive disorder: a proof-of-concept study. *Mol Psychiatry*. Oct 2015;20(10):1205-11. doi:10.1038/mp.2014.154
2. Lopes AC, Greenberg BD, Canteras MM, et al. Gamma ventral capsulotomy for obsessive-compulsive disorder: a randomized clinical trial. *JAMA Psychiatry*. Sep 2014;71(9):1066-76. doi:10.1001/jamapsychiatry.2014.1193
3. Yin D, Zhang C, Lv Q, et al. Dissociable Frontostriatal Connectivity: Mechanism and Predictor of the Clinical Efficacy of Capsulotomy in Obsessive-Compulsive Disorder. *Biol Psychiatry*. Dec 15 2018;84(12):926-936. doi:10.1016/j.biopsych.2018.04.006

Author contributions

Y-J.Z., L.M., W.L., B.S., and V.V. contributed to the conception and design of the data. Y-J.Z., H.C., Y.Z., R.C., H.L., J.Z., W.L. and B.S. contributed to acquisition of the data. Y-J.Z. and R.C. analyzed the data. Y-J.Z., H.C., drafted the manuscript. Y-J.Z., H.C., W.L., and V.V interpreted the results. All authors contributed to the edition of the manuscript.
